# Sulfur Switches for Responsive Peptide Materials

**DOI:** 10.1021/acs.accounts.3c00626

**Published:** 2024-02-19

**Authors:** Timothy J. Deming

**Affiliations:** Department of Bioengineering, University of California, Los Angeles, California 90095, United States; Department of Chemistry and Biochemistry, University of California, Los Angeles, California 90095, United States

## Abstract

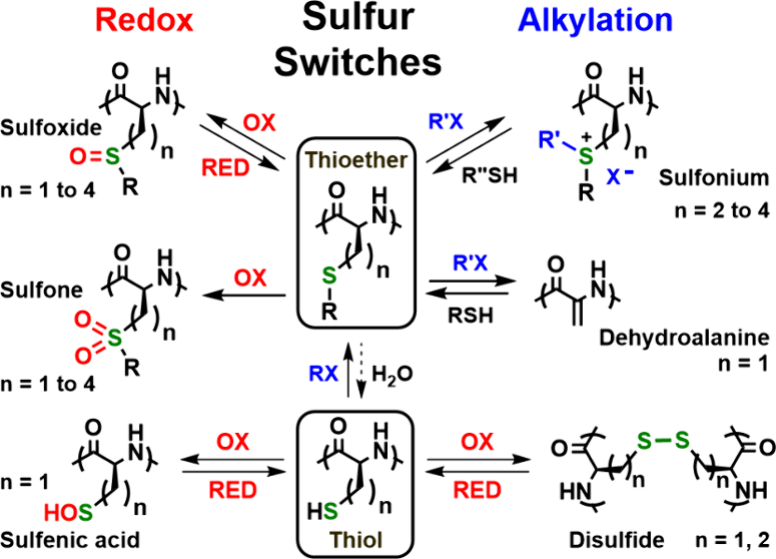

There is considerable recent interest in the synthesis and development of peptide-based materials as mimics of natural biological assemblies that utilize proteins and peptides to form organized structures and develop beneficial properties. Due to their potential compatibility with living organisms, synthetic peptide materials are also being developed for applications such as cell grafting, therapeutic delivery, and implantable diagnostic devices. One desirable feature for such applications is the ability to design materials that can respond to stimuli by changes in their structure or properties under biologically relevant conditions. Peptide and protein assemblies can respond to stimuli, such as changes in temperature, solution pH, ions present in media, or interactions with other biomacromolecules. An exciting area of emerging research is focused on how biology uses the chemistry of sulfur-containing amino acids as a means to regulate biological processes. These concepts have been utilized and expanded in recent years to enable the development of peptide materials with readily switchable properties.

The incorporation of sulfur atoms in polypeptides, peptides, and proteins provides unique sites that can be used to alter the physical and biological properties of these materials. Sulfur-containing amino acid residues, most often cysteine and methionine, are able to undergo a variety of selective chemical and enzyme-mediated reactions, which can be broadly characterized as redox or alkylation processes. These reactions often proceed under physiologically relevant conditions, can be reversible, and are significant in that they can alter residue polarity as well as conformations of peptide chains. These sulfur-based reactions are able to switch molecular and macromolecular properties of peptides and proteins in living systems and recently have been applied to synthetic peptide materials. Naturally occurring “sulfur switches” can be reversible or irreversible and are often triggered by enzymatic activity. Sulfur switches in peptide materials can also be triggered *in vitro* using oxidation/reduction and alkylation as well as photochemical reactions. The application of sulfur switches to peptide materials has greatly expanded the scope of these switches due to the ability to readily incorporate a wide variety of noncanonical sulfur-containing synthetic amino acids.

Sulfur switches have been shown to provide considerable potential to reversibly alter peptide material properties under mild physiologically relevant conditions. An important molecular feature of sulfur-containing amino acid residues was found to be the location of sulfur atoms in the side chains. The variation of sulfur atom positions from the backbone by single bond lengths was found to significantly affect polypeptide chain conformations upon oxidation–reduction or alkylation/dealkylation reactions. With the successful adaptation of sulfur switches to peptide materials, future studies can explore how these switches affect how these materials interact with biological systems. This Account provides an overview of the different types of sulfur switch reactions found in biology and their properties and the elaboration of these switches in synthetic systems with a focus on recent developments and applications of reversible sulfur switches in peptide materials.

## Key References

- RodriguezA. R.; KramerJ. R.; DemingT. J.Enzyme-triggered cargo release from methionine sulfoxide containing copolypeptide vesicles. Biomacromolecules2013, 14, 3610–3614.10.1021/bm400971p.23980867 (1) This study reports the use of methionine redox switching to drive a reversible morphology transition in polypeptide assemblies.
- KramerJ. R.; DemingT. J.Multimodal switching of conformation and solubility in homocysteine derived polypeptides. J. Am. Chem. Soc.2014, 136, 5547–555010.1021/ja500372u.24694061 (2) This work shows how redox and alkylation switches can independently and reversibly interconvert soluble α-helical and disordered homopolypeptide chain conformations in water.
- ScottW. A.; GharakhanianE. G.; BellA. G.; EvansD.; BahrunE.; HoukK. N.; DemingT. J.Active controlled and tunable coacervation using side-chain functional α-helical homopolypeptides. J. Am. Chem. Soc.2021, 143, 18196–1820310.1021/jacs.1c07925.34669392 (3) Side-chain functionality was used to replicate the pH-, ion-, and temperature-responsive properties of condensate-forming proteins using synthetic homopolypeptides that also contain embedded reversible on/off thioether redox switches.
- BenavidesI.; RafteryE. D.; BellA. G.; EvansD.; ScottW. A.; HoukK. N.; DemingT. J.Poly(dehydroalanine): synthesis, properties and functional diversification of a fluorescent polypeptide. J. Am. Chem. Soc.2022, 144, 4214–422310.1021/jacs.2c00383.35224969 (4) In this study, a new type of sulfur switch was developed where poly(*S*-alkyl-cysteine) and poly(dehydroalanine) are reversibly interconverted in aqueous media, which allows switching of both water solubility and chain conformations.

## 1 Introduction

The presence of sulfur atoms in peptides and proteins provides a means to switch their structure and/or function via enzymatic action or through the presence of reactive oxygen species (ROS) or reactive electrophile species (RES).^5,6^ There are many examples of natural “sulfur switches”, and these play significant and diverse roles in biology.^7^ Different types of sulfur switches also exist, and the identification of new switches and functions is an ongoing effort. The best characterized classes of sulfur switches include those that toggle between active and inactive states, e.g., on/off switches of enzyme activity.^5,6^ Allosteric switches regulate enzymes by adjusting their activity. Interaction switches change the binding properties of proteins, which is important in cell signaling. Finally, modification switches can completely change the function of a protein, e.g., the binding affinity for different molecules.^5,6^ The utility of sulfur switches is enhanced by the facile reversibility of chemical modifications. While many sulfur switches are reversible, there are also some that are not.^5−7^

**Figure 1:**
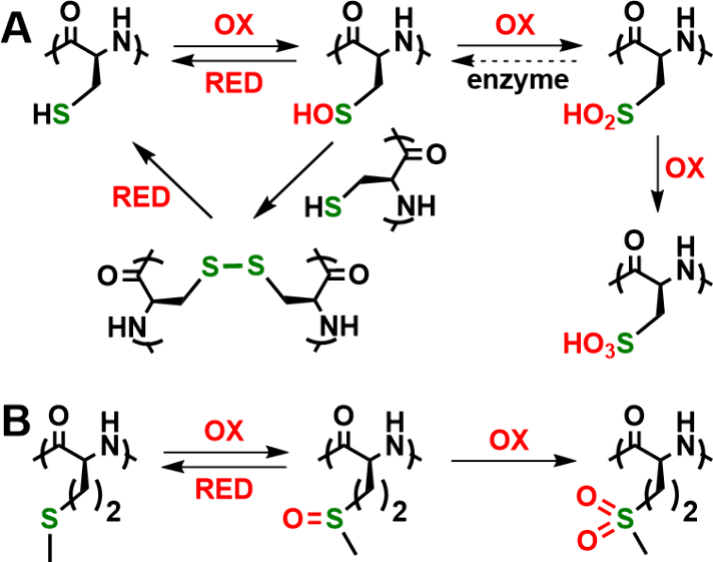
Oxidation reactions of natural sulfur containing (A) cysteine and (B) methionine residues in peptides and proteins. OX = oxidation; RED = reduction.

Numerous sulfur switches are driven by redox reactions.^8,9^ Since cysteine (Cys), methionine (Met), and less common selenomethionine (SeMet) are the only amino acids capable of reversible redox reactions in biological systems, they are excellent candidates for use as switches. The most widely recognized sulfur switch is the reversible redox interconversion of Cys thiols and cystine disulfides (Figure 1A).^5−7^ Disulfides are often formed between two Cys residues in the same peptide chain but can also occur between peptidic Cys and a small-molecule thiol such as glutathione.^5,6^ Cys residues are initially oxidized to sulfenic acid (sulfenylation), which is a reversible process that can switch the functional activity in proteins. Further oxidation of Cys sulfenic acids to sulfinic and sulfonic acids does also occur in biology (Figure 1A) as well as during oxidative stress with ROS. These reactions are generally considered to be not reversible, except for a few enzyme-mediated examples.^5−7^ The functional role of different levels of Cys oxidation is currently of great interest, especially in consideration of how oxidative stress acts on the proteome and how this can be mitigated.^5−7^ Met oxidation is less studied than Cys, but it is estimated that 5 to 10% of the Met proteome *in vivo* is present as Met sulfoxide (Figure 1B).^5,6,9^ Further oxidation to Met sulfone generally does not occur *in vivo*. It has been found that reversible oxidation of surface-exposed Met residues in proteins can act as switches to regulate enzymatic activity as well as drive phase separation.^8,9^

**Figure 2:**
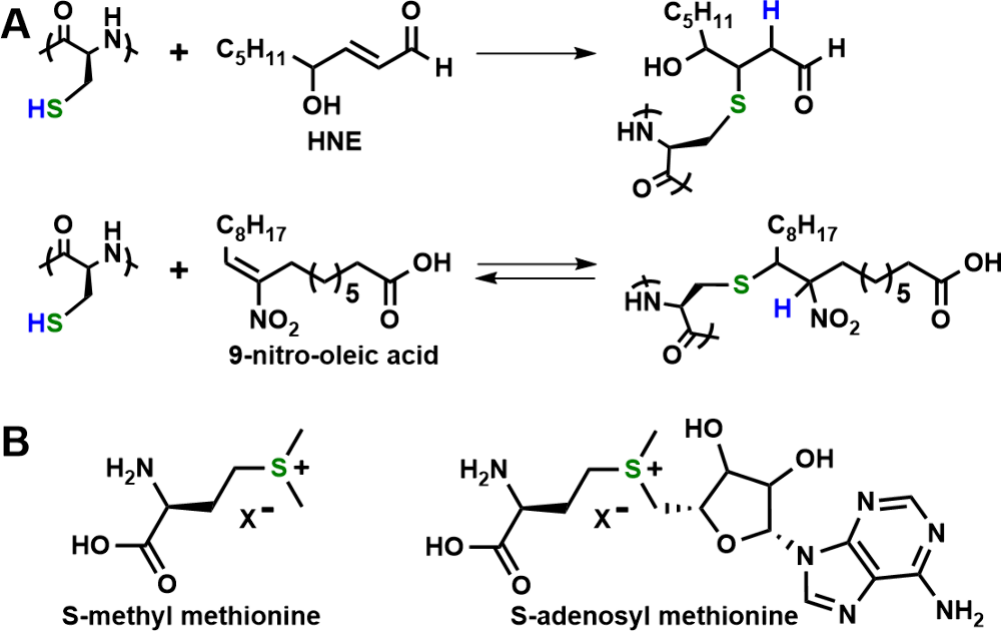
Biological alkylation reactions and products for natural sulfur containing (A) cysteine and (B) methionine residues in peptides and proteins. HNE = (*E*)-4-hydroxynon-2-enal.

Alkylation is another process employed in sulfur switches. Nucleophilic thiol groups in Cys are readily alkylated by a variety of endogenous RES that are produced in living systems (Figure 2A). Common RES include formaldehyde as well as electrophilic unsaturated carbonyl compounds, e.g., (*E*)-4-hydroxynon-2-enal (HNE), that are derived from the action of ROS on lipids.^7^ With molecules such as HNE, the thia-Michael addition of Cys thiol is essentially irreversible in a biological environment. However, the alkylation of Cys with endogenous nitroalkene RES is reversible via hydrolysis and has been found in some cases to act as an off/on switch of protein activity.^7^ While the selectivity of Cys alkylation is not fully understood, molecular specificity has been found in the reactivity of different RES with Cys thiols, and the treatment of hepatocytes with different enantiomers of HNE resulted in different phenotypic effects.^7^ Met can also react with RES, with the most notable example being the enzymatic formation of *S*-adenosyl-Met and *S*-methyl-Met sulfonium ions (Figure 2B), which are important cosubstrates in enzyme-mediated methyl group transfer reactions in living systems.^10,11^ Met alkylations tend to be reversible since the sulfonium ion products are themselves electrophilic. Overall, oxidation and alkylation reactions are the dominant processes that enable sulfur switches in biology.^8,9^ These switches have many functions and make up a considerable part of the proteome. Here, we show how sulfur switches can be adapted and used for the development of environmentally responsive peptide materials. Due to their added versatility and potential for biological applications, particular focus is placed on switches that can be reversed under physiologically relevant conditions.

## 2 Thiol Switches in Peptide Materials

**Figure 3:**
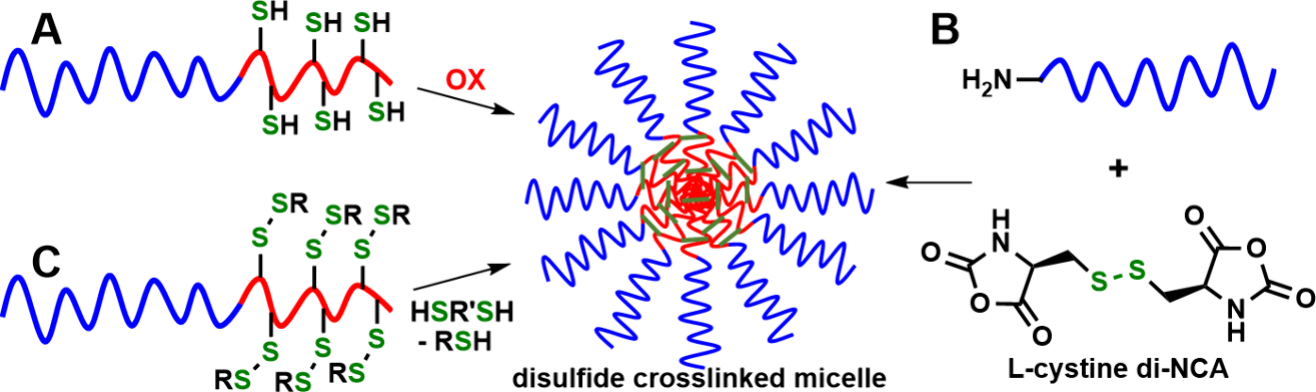
Different approaches for the preparation of cystine disulfide core cross-linked polymer micelles. (A) Oxidation of poly(l-cysteine) segments in amphiphilic block copolymers. (B) Direct cross-link formation by the polymerization of cystine di-NCA using hydrophilic macroinitiators. (C) Reaction of disulfide-protected poly(l-cysteine) segments in amphiphilic block copolymers with small-molecule dithiols. Blue chains = hydrophilic polymer. Red chains = cysteine/cystine-containing polymer.

The high reactivity of thiols under mild conditions relative to other peptide side-chain functional groups makes them attractive candidates for use in switchable peptide materials. While thiols can readily undergo both oxidation and alkylation reactions, the oxidation of thiols to disulfides has been the most widely used due to facile reversibility. In peptide materials, reversible disulfide bond formation is primarily used to switch between monomeric chains and multichain aggregates in aqueous media. Such reversible redox-switched formation and dissociation of peptide assemblies has been used extensively in the development of peptide micelles and hydrogels for therapeutic delivery and biomaterial applications. Different strategies have been employed to prepare disulfide cross-linked micelles (Figure 3). Our group prepared poly(l-lysine)-*block*-poly(l-cysteine), poly(Lys)-poly(Cys), linear copolymers, which formed aggregates in water after Cys thiol groups were allowed to oxidize to disulfide bonds in air (Figure 3A).^12^ These assemblies were able to catalyze silica formation giving micrometer-sized silica particles that varied from spheres to columns as the Cys to Lys ratio was increased.^12^ Qiao used a different approach by preparing poly(l-lysine)-*block*-poly(l,l-cystine) star copolymers, where disulfide cross-links were formed directly during polymer synthesis (Figure 3B).^13^ Spherical assemblies were formed with diameters ranging from *ca*. 50 to 100 nm and could be used to encapsulate water-insoluble drugs. Since not all *N*-carboxyanhydride (NCA) groups of cystine di-NCA were consumed to form cross-links during polymerization, residual NCAs in the micelle core could also be functionalized by postpolymerization reactions with primary amines.^13^

In a related study, Chen prepared micellar “nanogels” by the copolymerization of lysine or glutamic acid NCA with cystine di-NCA using a polyethylene glycol amine, PEG-NH_2_, macroinitiator.^14^ As above, disulfide cross-links were formed during copolymerization. Upon removal of the lysine or glutamate protecting groups, the resulting charged residues gave micelles with hydrated nanogel cores that could be loaded with doxorubicin. Reduction of the disulfide cross-links using glutathione resulted in dissolution of the assemblies and the release of doxorubicin. Recently, Barz reported another approach to disulfide cross-linked micelles based on poly(sarcosine)-*block*-(cysteine or homocysteine) polymers, poly(Sar)-poly(Cys) or poly(Sar)-poly(Hcy), where the Cys and Hcy thiol groups were protected as *S*-ethylsulfonyl disulfides (Figure 3C).^15,16^ Here, disulfide cross-linked micelles were obtained by the addition of small-molecule dithiols, which undergo disulfide exchange with protected Cys and Hcy residues. The use of Cys or Hcy gave uniform, nanoscale micelles (*ca*. 30 to 70 nm diameter) that were elongated or spherical, respectively, which was attributed to differences in chain conformations and the consequent aggregation of Cys (β-sheet) and Hcy (α-helical) segments. Poly(Sar)-poly(Cys) micelles with hydrophobic cross-linkers were used to encapsulate the drug paclitaxel, and micelles with cationic cross-linkers were used to encapsulate siRNA. Reduction of disulfides using glutathione resulted in micelle disruption and release of the siRNA. This route to disulfide cross-linked peptide micelles appears to be the most promising as micelles can be formed using soluble and stable precursors, and cross-linking can be performed using a controllable process that can independently add functionality or change the polarity of the micelle core.

**Figure 4:**
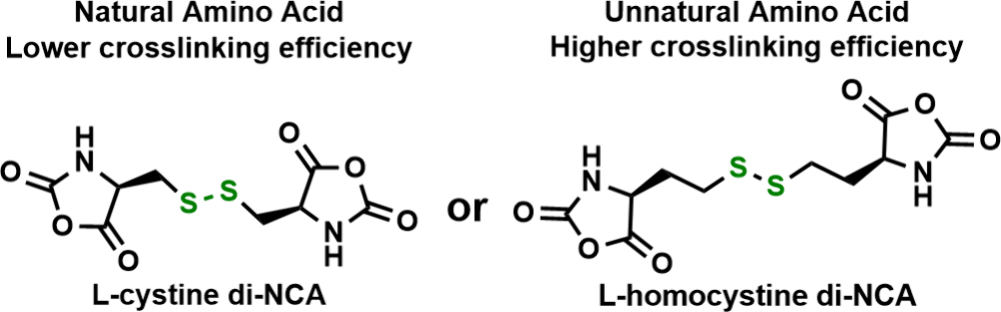
Comparison of l-cystine di-NCA and l-homocystine di-NCA.

Aside from micelles, peptide hydrogels have also been prepared using disulfide cross-linking. Chilkoti’s laboratory prepared elastin-like protein (ELP) sequences containing periodic cysteine residues. Upon oxidation with H_2_O_2_ in water, disulfide cross-links form, resulting in bulk hydrogel networks.^17^ These hydrogels showed prolonged release of a model protein cargo (albumin), and an injectable formulation was developed that can form hydrogel deposits *in vivo*. Our group prepared synthetic polypeptide hydrogels directly via statistical copolymerization of *tert*-butyl-glutamate NCA and either cystine or homocystine di-NCAs, followed by the removal of *tert*-butyl protecting groups (Figure 4).^18^ Reduction of disulfide bonds using tris-carboxyethylphosphine resulted in hydrogel dissolution. Comparison of cystine versus homocystine cross-linkers showed that homocystine di-NCA, with its longer tether, was significantly more efficient at forming interchain cross-links during copolymerization compared to cystine di-NCA. The cross-linking efficiency was gauged by a comparison of hydrogel stiffness at equivalent cross-linker feeds, where the Hcy gels were *ca*. 10 times more stiff compared to Cys gels.

**Figure 5:**
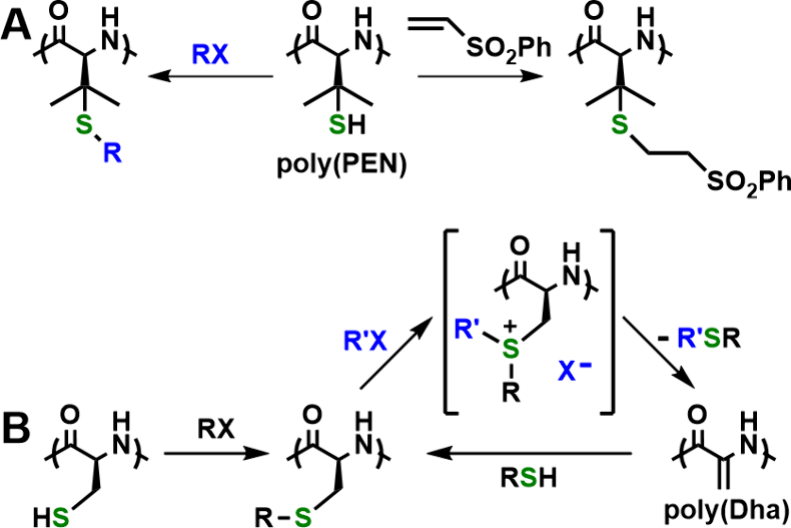
Alkylation reactions of cysteine and cysteine mimetic residues in peptide materials. (A) Irreversible alkylations of poly(penicillamine), poly(PEN). (B) Alkylation of poly(cysteine) followed by reversible interconversion of poly(alkyl-cysteine) and poly(dehydroalanine), poly(Dha).

In addition to redox, peptide materials can also be switched by the alkylation of thiols. Nucleophilic Cys residues in peptides and polypeptides are readily, and often selectively, alkylated under mild conditions using electrophilic reagents such as alkyl halides. While such modifications allow for switching peptide properties by the introduction of functional groups, such as sugars, these reactions require deprotection of Cys residues before alkylation as well as precautions to avoid thiol oxidation.^19^ A noteworthy exception is the thiol groups in poly(penicillamine), poly(PEN), which do not require protection, and their alkylations proceed in high yield since the tertiary thiols are less prone to oxidation (Figure 5A).^20^ While the alkylation of Cys residues in peptide materials is useful for adding functionality, it is not widely used as a switch since generally the alkylation reaction is irreversible.

To add to these accomplishments, our group recently developed a strategy to reversibly switch poly(Cys) properties via double alkylation of thiol groups.^4^ It is known that the double alkylation of isolated Cys residues in peptides and proteins generates sulfonium ions that readily eliminate thioethers to give unsaturated dehydroalanine (Dha) residues (Figure 5B). The addition of small-molecule thiols to electrophilic Dha results in the formation of alkyl-Cys residues, similar to those prepared by direct alkylation of Cys as described above. We have shown that a water-soluble poly(carboxymethylcysteine) poly(CMC) precursor can be alkylated in a similar manner to give hydrophobic poly(Dha). Subsequent addition of mercaptoacetic acid to poly(Dha) regenerates the poly(CMC) precursor.^4^ This interconversion of poly(CMC) and poly(Dha) is reversible in aqueous media and allows switching of both water solubility and chain conformation via thiol alkylation reactions.

## 3 Thioether Redox Switches

Thioether functional groups primarily exist in peptides and proteins as natural Met residues but can also be formed by the alkylation of Cys thiol groups as described above. Similar to thiols, thioether groups in Met are readily oxidized to methionine sulfoxide (MetO) (Figure 1). MetO is reduced by the action of methionine sulfoxide reductase enzymes that are ubiquitous in living cells, allowing the redox properties of Met to be fully reversible *in vivo*.^9^ MetO residues in peptide materials can also be readily reduced to Met using mild chemical reducing agents (e.g., thiols) in aqueous media. It is possible to further oxidize peptidic sulfoxides to sulfones using stronger oxidizing conditions, but this does not occur *in vivo* and is also an irreversible reaction. Thioether oxidation provides a useful, and often reversible, switch in peptide materials since it has been found to strongly affect peptide chain conformations as well as polarity.

The oxidation of Met in proteins has been under investigation for some time.^9^ MetO formation can serve as a reversible regulatory switch and can also function as a sacrificial process to consume ROS in a nondestructive manner while preserving more sensitive protein functionality. In peptides, Gellman reported an 18 residue sequence designed with periodically spaced Met residues that was studied in water in both reduced and oxidized forms.^21,22^ The Met residues were chosen since they can be reversibly switched between hydrophobic (thioether) and hydrophilic (sulfoxide) states. When hydrophobic, the peptide was α-helical, and when oxidized, the peptide formed β-strands, as designed. García-Echeverría also showed that homodimeric peptide coiled coils could be reversibly dissociated when single Met residues in the sequences were oxidized to sulfoxides.^23^ Switching of the phase-separation temperature in ELPs was also demonstrated by Deming and Lecommandoux by the incorporation and oxidation of periodic Met guest residues in ELP sequences.^24^ These studies on isolated Met residues in peptide and protein sequences show that the significant change in polarity between Met and MetO can enable a potent, reversible switch of the physical properties.

**Figure 6:**
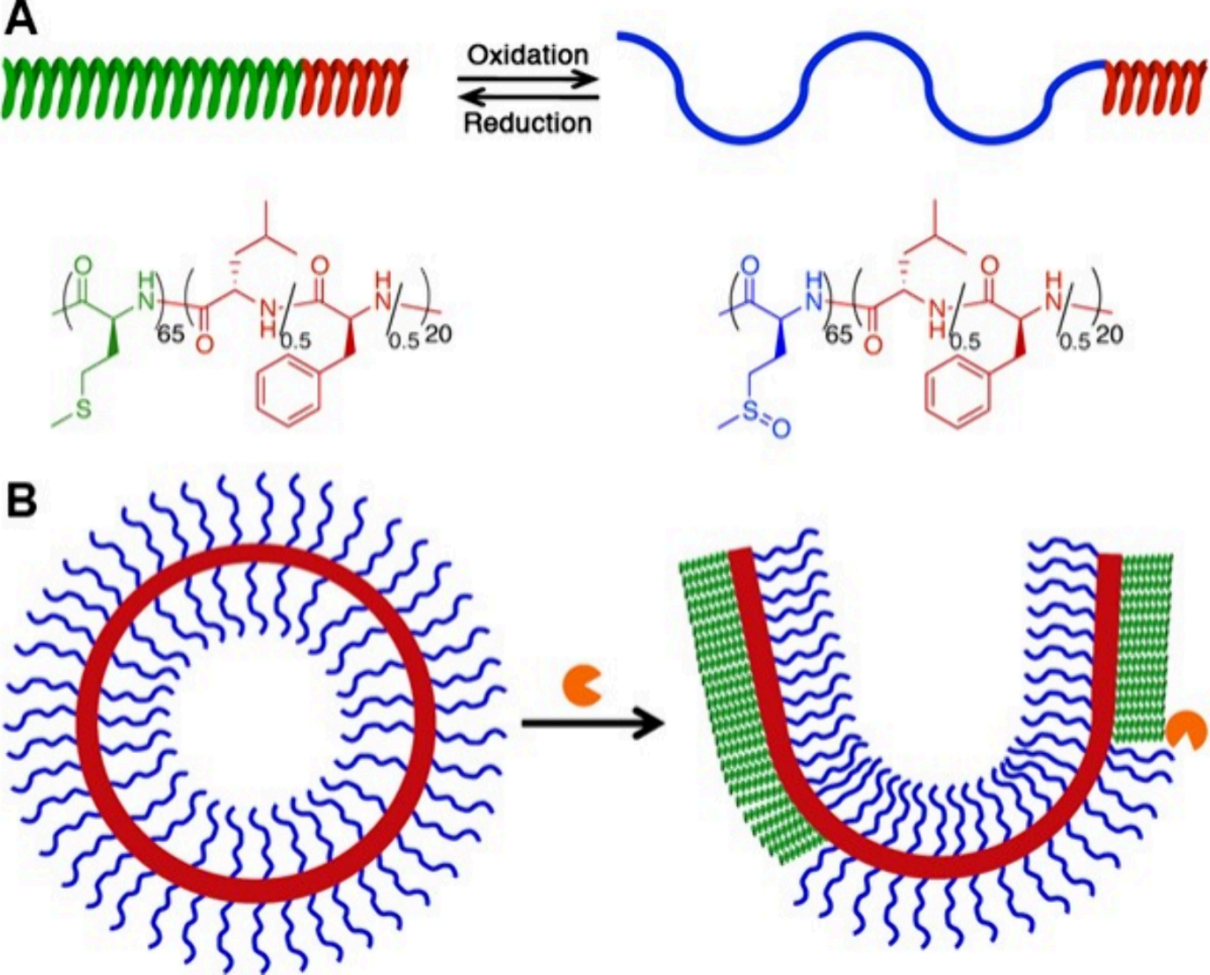
(A) Schematic showing the structure, chain conformations, and reversible redox properties of poly(MetO)_60_-*block*-poly(Leu/Phe)_20_. (B) Schematic showing how the enzymatic reduction of vesicle surface MetO segments to Met segments can result in vesicle disruption. Adapted with permission from ref (1). Copyright 2013 American Chemical Society.

Aiba was the first to report the properties of poly(l-methionine sulfoxide), poly(MetO), which was found to be soluble and to possess a disordered conformation in water.^25^ Poly(Met) is a hydrophobic α-helical polypeptide with poor water solubility and has been known since the late 1950s.^26,27^ More recently, our group reinvestigated the oxidation of polyMet, confirming the properties of the thioether and sulfoxide forms and also determining that poly(l-methionine sulfone), poly(MetO_2_), is α-helical with poor solubility in water.^1^ The switchable properties of Met were used by our group to prepare enzyme-responsive polypeptide assemblies (Figure 6). A fully hydrophobic diblock copolypeptide, poly(Met)_60_-*block*-poly(Leu/Phe)_20_, was prepared and then oxidized with H_2_O_2_ to switch it to the amphiphilic sulfoxide derivative poly(MetO)_60_-*block*-poly(Leu/Phe)_20_.^1^ In water, the α-helical poly(Leu/Phe) segments drove the assembly of the polypeptides into microscopic vesicles, which could be extruded to diameters of *ca*. 100 nm. Addition of methionine sulfoxide reductases, as are found in the cell cytosol, to the vesicle suspension resulted in a reversal of the oxidation switch to regenerate hydrophobic Met residues. The formation of α-helical, hydrophobic poly(Met) drove a structural change from vesicles to sheets that also ruptured the vesicles and released a model fluorescent dextran cargo (Figure 6).^1^ While promising for therapeutic delivery applications, this was also the first use of a sulfur switch to drive a reversible morphology transition in polypeptide assemblies.

Chen used the switchable properties of Met to make hydrogels that were designed to be degraded by ROS *in vivo*.^28^ They prepared PEG-*block*-poly(Met)_*n*_, *n* = 10, 14, 20, copolymers that formed micellar suspensions in water. Heating of the samples between 20 and 60 °C at *ca*. 6 to 10 wt % in aqueous phosphate buffer resulted in the Met segments adopting β-strand conformations, which lead to the formation of translucent hydrogels. Oxidation of the samples by the addition of H_2_O_2_ resulted in hydrogel erosion and dissolution due to the disruption of β-sheets by the introduction of polar MetO and MetO_2_ residues. A Rhodamine 6G model cargo was released from the hydrogels at rates that increased with the H_2_O_2_ concentrations. Recently, Battaglia prepared similar PEG-*block*-poly(Met)_*n*_, *n* = 5 to 120, copolymers where some samples possessed longer Met segments that favor stable α-helical conformations.^29^ Short Met segments gave spherical micelle assemblies in water, intermediate lengths gave wormlike micelles, and long segments gave nanoscale vesicles. Use of H_2_O_2_ as an oxidation switch, as a mimic of ROS exposure, resulted in dissolution of the assemblies. Overall, poly(Met) and poly(MetO) chains show great promise for use as biomaterials that incorporate natural amino acids and can be reversibly switched at physiologically relevant ROS concentrations or via the action of intracellular enzymes.

**Figure 7:**
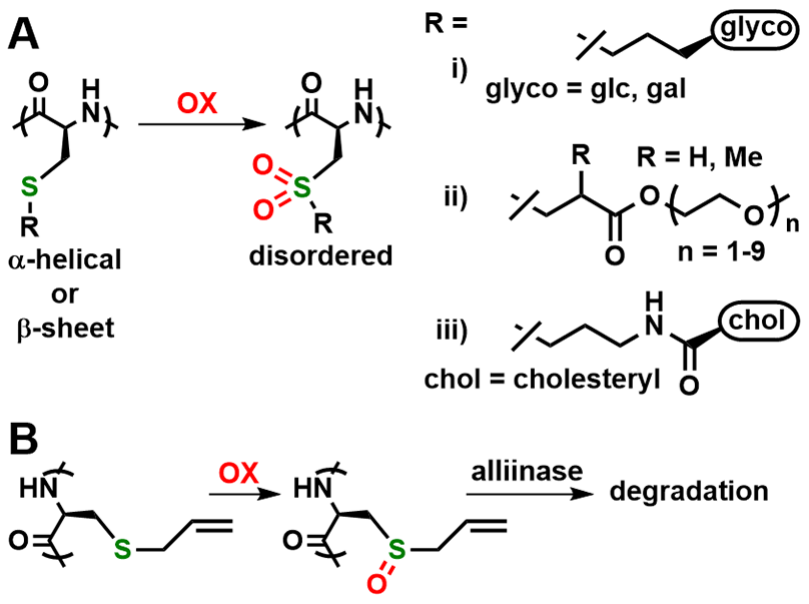
Alkylation reactions of alkyl-cysteine residues in peptide materials. (A) Irreversible oxidation to sulfones results in the disruption of ordered chain conformations. glc = α-d-glucopyranoside; gal = α-d-galactopyranoside. (B) Reversible oxidation of poly(allyl-l-cysteine) to give poly(alliin) and its subsequent degradation using the enzyme alliinase.

Although not as thoroughly investigated as Met, thioethers containing alkylated Cys residues have also been oxidized in synthetic peptide materials as a means to switch their properties. Our group showed that water-soluble, α-helical glycosylated poly(*S*-alkyl-l-cysteine)s, poly(R-Cys), underwent an irreversible switch to disordered conformations when oxidized to the sulfone derivatives (Figure 7A).^30^ In these polymers, where the bulky pendant sugar groups favor α-helical conformations, reversible oxidation to the sulfoxides resulted in a negligible conformational change. Li subsequently reported that hydrophilic oligoethylene glycol (OEG) alkylated poly(R-Cys), poly(OEG-Cys), also undergoes irreversible conformational changes from β-sheet to disordered upon oxidation to sulfone derivatives (Figure 7A).^31^ Block copolymers of these poly(OEG-Cys) segments with PEG were used to form doxorubicin-loaded micelles in water that could then be switched to dissolve upon oxidation with H_2_O_2_ and release the drug. In a related study, Ding prepared PEG-*block*-poly(R-Cys) containing hydrophobic cholesteryl groups in the side chains (Figure 7A).^32^ The polypeptide segments formed β-sheets, and the assembly of these amphiphiles in water gave stable micelles. Oxidation of thioether groups to sulfones switched the polypeptide conformations, which resulted in an irreversible transition of the assemblies from micelles to vesicles.

Oxidation of poly(R-Cys) to sulfoxides has also been found to switch polypeptide properties in some cases. Bonduelle found that water-soluble, disordered poly(carboxyethyl-l-cysteine) adopted a β-sheet conformation when complexed with Cd^2+^ ions in water at pH 6.5 and that mild oxidation of the polymer to the sulfoxide resulted in a switch to a disordered conformation.^33^ Limiting the R-Cys thioether oxidation to the sulfoxide gave a switch that was reversible under mild conditions. Ling also recently reported a reversible switch based on poly(*S*-allyl-l-cysteine).^34^ This polymer is hydrophobic and forms β-sheets, but when oxidized to the sulfoxide, the resulting poly(alliin) was water-soluble and was also amenable to specific degradation with the enzyme alliinase (Figure 7B). Block copolymers of PEG with poly(*S*-allyl-l-cysteine) formed micelles in water that dissolved upon oxidation with H_2_O_2_. It is encouraging that reversible sulfur switches are beginning to be developed with poly(R-Cys). The variety of alkyl groups and other functionalities that can be conjugated to Cys residues in polypeptides provides many opportunities for further development.

**Figure 8:**
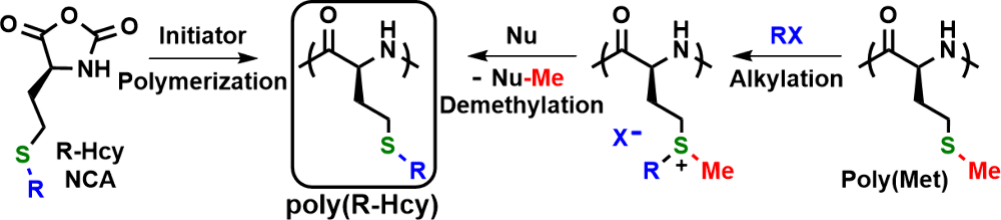
Preparation of poly(alkyl-l-homocysteine)s, poly(R-Hcy), from either R-Hcy NCA polymerization or the alkylation and demethylation of poly(l-methionine), poly(Met).

**Figure 9:**
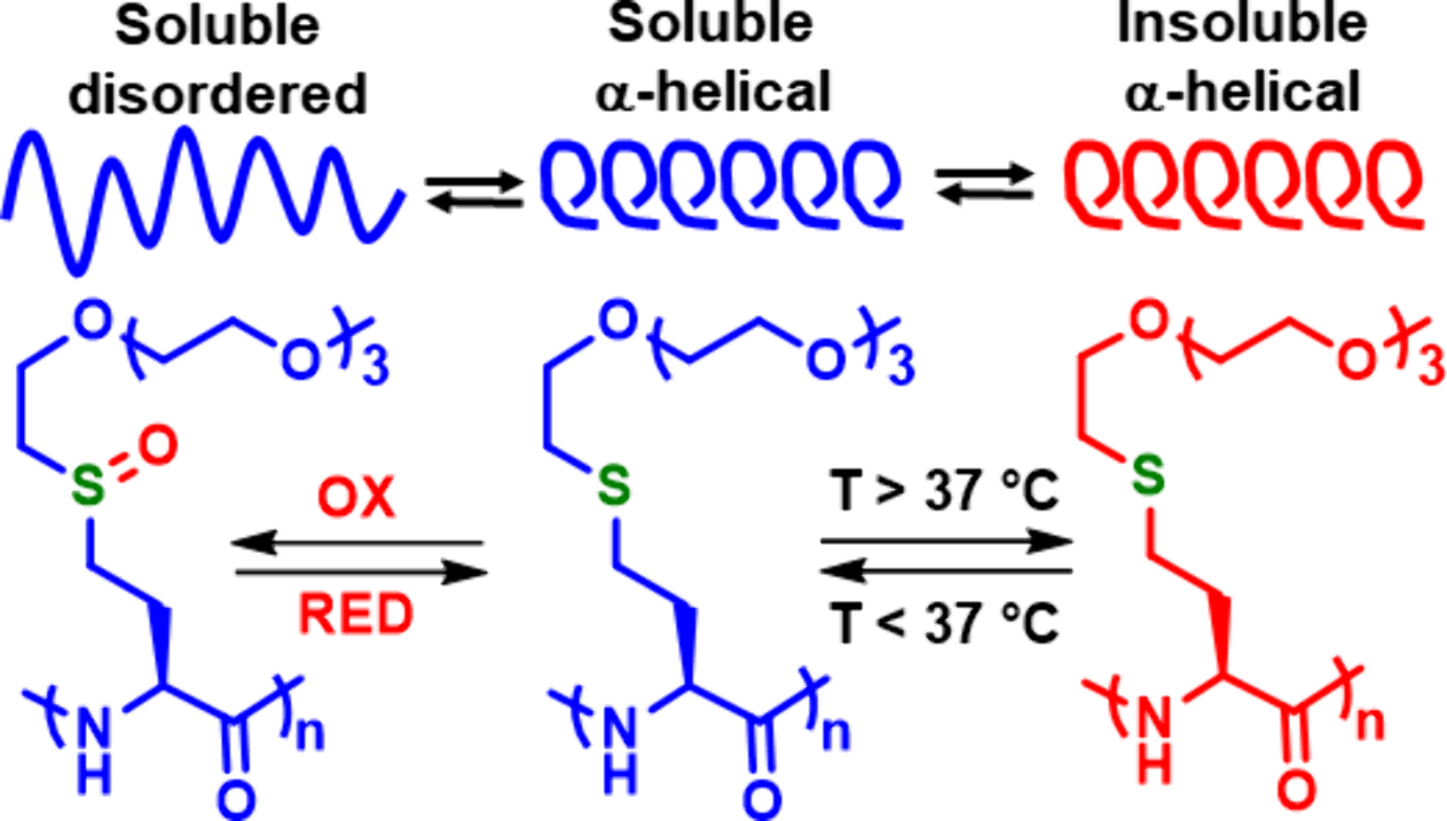
Reversible and independent multimodal switching of chain conformation and water solubility with poly(2,5,8,11-tetraoxatridecyl-l-homocysteine), poly(EG_4_-Hcy).

In addition to Met and Cys, there has been significant recent effort in the development of switchable peptides using other thioether-containing amino acids. One important class is poly(alkyl-l-homocysteine)s, poly(R-Hcy), which can be prepared from alkylated Hcy amino acids or by postpolymerization modification of poly(Met) (Figure 8). Since the alkyl (R) group can be readily varied, it is possible to adjust the polypeptide solubility or functionality independently of the thioether sulfur switch. Using this concept, the first fully reversible switching of homopolypeptide chain conformations in water, where both α-helix and disordered conformations remain soluble, was achieved by reversible oxidation of the OEG-functionalized poly(EG_4_-Hcy) (Figure 9).^2^ In reduced form, this polypeptide adopts a stable α-helical conformation similar to that of poly(Met), and when oxidized to the sulfoxide, poly(EG_4_-HcyO), the polypeptide similarly switches to a disordered conformation (Figure 9). The key difference from poly(Met) is that the EG_4_ substituents provide water solubility for both the reduced and oxidized forms. The change in conformation upon oxidation also serves as a switch for temperature-responsive properties, since poly(EG_4_-Hcy) phase separates from water at *ca*. 37 °C, while poly(EG_4_-HcyO) remains soluble up to 90 °C. This ability to reversibly switch multiple polypeptide properties by design is a powerful feature found in poly(R-Hcy) that surpasses what is possible with Met and R-Cys polymers.^2^ Recently, the Lu group reported analogous OEG alkylated poly(alkyl-l-selenohomocysteines), e.g., poly(EG_4_-SeHcy), that possess solution properties similar to those of poly(EG_4_-Hcy) but can be reversibly oxidized under very mild conditions.^35^ Poly(EG_4_-SeHcy) segments were used in assemblies that could undergo oxidative switching due to ROS in cells to change from amorphous to fibril morphologies and consequently release doxorubicin.^36^

**Figure 10:**
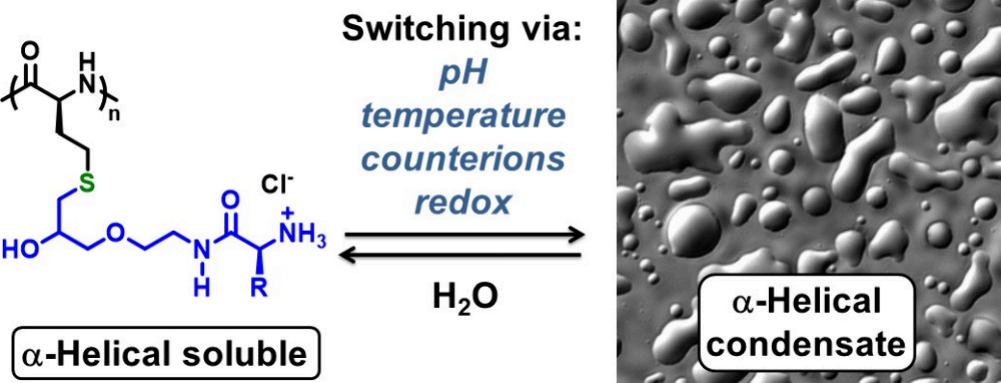
α-Helical amino acid containing poly(R-Hcy) that can reversibly switch between soluble and phase-separated condensate states via a variety of physiologically relevant stimuli. Adapted with permission from ref (3). Copyright 2021 American Chemical Society.

To showcase the variety of properties that can be obtained by changing the R group in poly(R-Hcy), our group prepared a variety of OEG alkylated poly(R-Hcy) from poly(Met) via alkylation with epoxides followed by demethylation of the resulting sulfoniums.^37^ Due to the OEG groups, these polymers display temperature-dependent solubility in water, and the transition temperature can be varied from 28 to 76 °C by straightforward alteration of the OEG structures. Oxidation of all of these polymers to sulfoxides resulted in reversible switching to disordered conformations and loss of phase transitions. Irreversible oxidation to sulfones retained the α-helical conformations but switched off the phase transition. Oxidative switching in these polymers allows control over thermal properties independent of chain conformation. To go beyond switching of only thermal properties, our group introduced amino acid functionality into poly(R-Hcy) side chains (Figure 10).^3^ The incorporation of both hydrophobic and charged groups provided sensitivity to solution pH, ions present in media, and temperature. The goal of this work was to recreate properties of stimuli-responsive condensate-forming proteins using switchable synthetic homopolypeptides. Variation of side-chain groups gave polypeptides that could form condensates in water by changing the pH, ionic media, and temperature within physiological ranges, and these properties could be reversibly switched off and on by the oxidation of side-chain thioether groups to sulfoxides and then reversed by reduction (Figure 10).^3^

**Figure 11:**
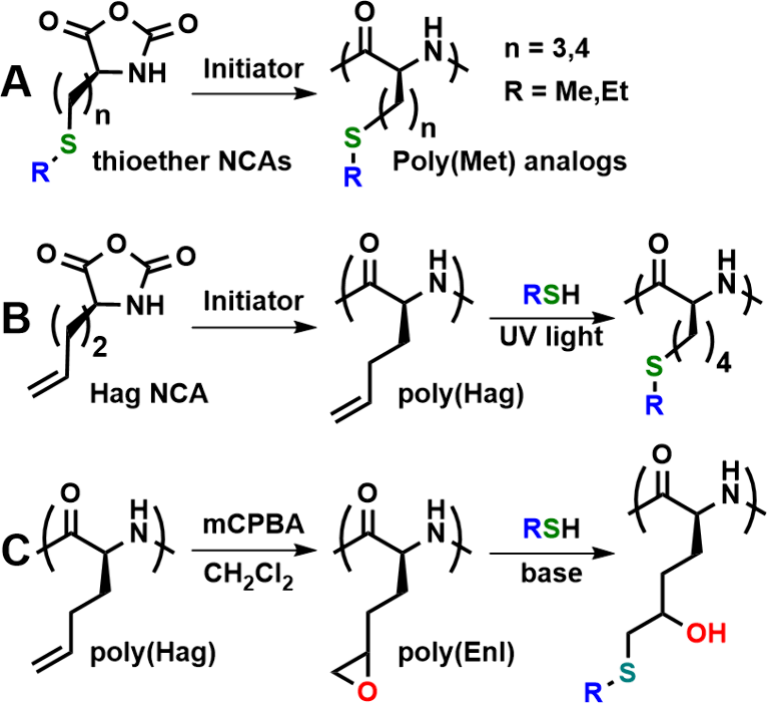
Preparation of poly(R-Hcy) analogs via different synthetic routes. (A) Direct polymerization of thioether containing NCA monomers. (B) Preparation of poly(l-homoallylglycine), poly(Hag), and conversion to thioether-containing polypeptides via thiol–ene reactions. (C) Oxidation of poly(Hag) to give poly(l-epoxynorleucine), poly(Enl), followed by reactions with nucleophilic thiols.

In addition to varying the alkyl groups in poly(R-Hcy), it is also possible to vary the spacing of thioether groups from the peptide backbone, as seen in poly(R-Cys) and poly(R-Hcy). Our group prepared a series of higher homologues of poly(R-Hcy) that contain three and four methylenes between the backbone and sulfur atoms (Figure 11A).^38^ Upon reversible oxidation of thioether groups to sulfoxide derivatives when R = Me, Et, or both, these polypeptides all undergo a solubility switch from hydrophobic to water-soluble. However, the increased distance of sulfoxide groups from the backbone in higher homologues allows one to tune out the conformational switch that occurs with poly(R-Hcy) such that the chains switch only between water-insoluble and water-soluble α-helical states. These samples were prepared by using synthetic amino acids, which required many steps for each derivative. We have shown that polypeptides with similar side-chain structures and a wider variety of terminal R groups can be more readily prepared via postpolymerization modification reactions. Soluble, readily prepared poly(l-homoallylglycine), poly(Hag), is readily converted by thiol–ene reactions with different thiols into a variety of poly(6-(alkylthio)-l-norleucine) derivatives (Figure 11B).^39^ With hydrophilic alkyl groups, such as the OEG, the water-soluble, α-helical polypeptides can be reversibly switched to partially disordered conformations via oxidation to sulfoxides. Another route employed involves the epoxidation of poly(Hag) to give poly(l-epoxynorleucine), poly(Enl), which reacts directly with thiols to give hydroxyl-substituted poly(6-(alkylthio)-l-norleucine)s (Figure 11C).^40^ A variety of thiols can be used here as well to make a range of derivatives, but the hydrophilic hydroxyl groups cause disorder in the chain conformations, resulting in little change in the conformation after oxidation to sulfoxides. Overall, the oxidation of thioether side chains to sulfoxides is a powerful means to reversibly switch the properties of a broad class of polypeptides. The recent introduction of biomimetic functionality into these switchable polypeptides,^3^ combined with the physiologically relevant oxidation conditions, is expected to lead to many useful applications of these sulfur switches.

## 4 Thioether Alkylation Switches

**Figure 12:**
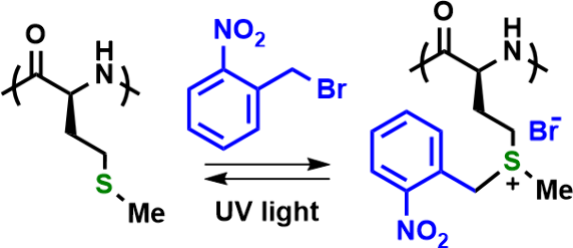
Reversible switching of poly(Met) properties via alkylation with *O*-nitrobenzyl bromide followed by the photochemical cleavage of *O*-nitrobenzyl groups.

Alkylation of Met residues in peptides and proteins has been studied for many decades and has historically been used to study its effects on biological activity.^10,11^ More recently, Met alkylation has been used as a means to add functional groups to these residues in polypeptides, peptides, and proteins. Reaction of poly(Met) with electrophilic alkylating agents (e.g., alkyl halides and epoxides) gives poly(*S*-alkyl-l-methionine), poly(R-Met^+^) sulfonium ions that are generally stable and can be isolated.^41,42^ Our group also developed a two-step poly(Met) alkylation and demethylation process that can conveniently use poly(Met) as an economical, universal precursor to a wide range of stable, functional poly(R-Hcy).^43^ To use Met alkylation as a sulfur switch, it is desirable for the alkylation reaction to give a stable sulfonium product but also to be reversible. Our group found that benzylic halides are useful reagents for the reversible alkylation of Met and R-Hcy residues.^44^ Alkylation proceeds under mild conditions (neutral or acidic water), and the sulfonium products are stable for extended periods in phosphate buffer. Addition of thiol, e.g., 2-mercaptopyridine, removes the benzyl groups and regenerates the Met or R-Hcy residues. Mandal has also reported *O*-nitrobenzyl alkylation of poly(Met), where the resulting sulfonium ions can be switched back to Met by photochemical removal of the *O*-nitrobenzyl groups, acting as a photoswitch (Figure 12).^45^ Benzylic alkylation switches have been used to reversibly modify peptides and ELPs and to reversibly switch poly(EG4-Hcy) conformations between α-helical and disordered states in water.^2,44,46^ This methodology is an alternative to sulfoxide oxidation for reversible switching, and it permits one to consider using different sulfur switches for different thioether-containing residues to perform more complex manipulations of properties.^39^

## 5 Outlook and Summary

There is considerable potential to reversibly alter peptide material properties under mild, physiologically relevant conditions via the incorporation of “sulfur switches”. An important feature of sulfur-containing amino acid residues that affects their switching properties is the precise location of the sulfur atom in the side-chain molecular structure. This is exemplified by Cys, Hcy, and beyond, where each addition of a methylene spacer significantly affects how chain conformations and reactivity are altered via redox or alkylation/dealkylation reactions. With the successful adaptation of sulfur switches to peptide materials, future directions of this field can explore how these switches can go beyond the alteration of physical and chemical properties to affect how peptide materials interact with biological systems.
